# Intensity of Humoral Immune Responses, Adverse Reactions, and Post-Vaccination Morbidity after Adenovirus Vector-Based and mRNA Anti-COVID-19 Vaccines

**DOI:** 10.3390/vaccines10081268

**Published:** 2022-08-06

**Authors:** Ioanna Voulgaridi, Styliani Sarrou, Aikaterini Dadouli, Athanasia-Marina Peristeri, Asimina Nasika, Ilias Onoufriadis, Maria A. Kyritsi, Lemonia Anagnostopoulos, Aikaterini Theodoridou, Ioanna Avakian, Dimitra Pappa, Adamos-Konstantinos Konstantinou, Georgia Papadamou, Varvara A. Mouchtouri, Efi Petinaki, Matthaios Speletas, Christos Hadjichristodoulou

**Affiliations:** 1 Laboratory of Hygiene and Epidemiology, Faculty of Medicine, University of Thessaly, 41222 Larissa, Greece; 2 Department of Immunology & Histocompatibility, Faculty of Medicine, University of Thessaly, 41500 Larissa, Greece; 3 Department of Pathology, Faculty of Medicine, University Hospital of Larissa, 41500 Larissa, Greece; 4 Psychogeriatric Hospital “Ippokrateio Therapeutirio”, 40011 Larissa, Greece; 5 Emergency Department, University Hospital of Larissa, 41500 Larissa, Greece; 6 Department of Microbiology, University Hospital of Larissa, 41500 Larissa, Greece

**Keywords:** COVID-19, vaccination, IgG, IgA, antibody responses, BNT162b2, Ad26.COV2.S, ChAdOx1 nCoV-19

## Abstract

The aim of the study was to compare mRNA vaccine BNT162b2 with adenovirus vector- based vaccines in terms of presence of adverse reactions, immunogenicity, and protection against COVID-19. A total of 270 individuals were enrolled, of which 135 were vaccinated with adenovirus vector-based vaccines and compared with 135 age- and sex-matched participants who received the BNT162b2 mRNA vaccine. Serum sampling was performed on all participants on days 21, 42, 90, and 180 following the first dose, to evaluate anti-spike IgG and IgA responses. Antibodies were quantified by chemiluminescent microplate and ELISA assays. We demonstrate that both mRNA and adenovirus vector-based vaccines caused mild side-effects and were effective in inducing adequate antibody responses against SARS-CoV-2, although BNT162b2 was superior concerning the intensity of antibody responses and protection against severe COVID-19. Moreover, we identify that IgG and IgA responses depended primarily on both history of previous COVID-19 infection and vaccination platform used, with individuals immunized with a single-dose vaccine having lower antibody titers over time. Lastly, all vaccine platforms had limited side-effects, with the most frequent pain at the injection site. Our results provide useful information regarding antibody responses after vaccination with different vaccine platforms, which can be useful for public health vaccination strategies.

## 1. Introduction

Soon after its emergence, coronavirus disease 2019 (COVID-19) became a global pandemic leading to a loss of human life, as well as destabilization of the global economy and public health systems [1,2,3]. The development of vaccines to prevent infection became an effective tool against the pandemic. Thus, different vaccine platforms were developed and received emergency use authorization from the Food and Drug Administration (FDA) and/or European Medicines Agency (EMA), leading to their wide use [4,5,6,7,8]. In Greece, vaccination against COVID-19 began in late December 2020 with BNT162b2 vaccine (Comirnaty^®^; BioNTech/Pfizer, Mainz, Germany); a few months later, the ChAdOx1 nCoV-19 (AZD1222 of Oxford/AstraZeneca, University of Oxford, UK) and Ad26.COV2.S (Janssen Biontech, Inc., Janssen Pharmaceutical company, Johnson & Johnson, New Brunswick, NJ, USA) vaccines were added to the vaccination campaign.

Severe acute respiratory syndrome coronavirus 2 (SARS-CoV-2) which causes COVID-19 is a positive-stranded RNA virus, and its genome includes multiple regions encoding nonstructural, accessory, and structural proteins such as spike (S), membrane (M), nucleocapsid (N), and envelope (E) proteins. The S protein, especially the receptor-binding domain (RBD) of its S1 subunit, is crucial for virus entry into host cells through binding to human angiotensin-converting enzyme 2 receptor (ACE2) that is located on cell membranes [9,10]. In addition, protein S plays an important role in the host’s immune response due to its high antigenicity and ability to induce immune response [10]. Patients after COVID-19 develop anti-S antibodies with neutralizing activity [11,12]. Vaccines developed for SARS-CoV-1 and MERS-CoV were based on protein S-inducing neutralizing antibodies with a protective effect [12]. Therefore, the S protein was considered as a competent immunodominant target for most SARS-CoV-2 vaccines. Thus, most of them are either based on the S protein or on its immunogenetic region. The desired outcome for COVID-19 vaccines is to produce antibodies that will prevent the entrance of the virus into the host cell, thereby inhibiting its replication. The produced neutralizing antibodies are associated with epitopes of the S protein, and it was found, in the serum of patients after COVID-19, that the RBD region was the primary target of these antibodies [12]. Part of the success of vaccines is also the ability to prevent and protect against infection from new variants that may be more virulent, pathogenic, or capable of avoiding immunity due to mutations on S protein. Although studies suggest that vaccines can protect against severe COVID-19 from circulating variants of concern [13], the effectiveness of currently used vaccines against them is under evaluation [12,13,14].

The BNT162b2 messenger ribonucleic acid (mRNA) vaccine is a lipid nanoparticle-formulated RNA, encoding the SARS-CoV-2 full-length S protein, modified by two proline mutations to lock it in the prefusion conformation [5,8,15,16]. It requires cold storage and is administered intramuscularly in two doses, with an interval of 21 days [16]. The ChAdOx1 nCoV-19 vaccine is a recombinant, replication-deficient chimpanzee adenovirus vector also encoding the S protein. The vaccine is indicated for active immunization of individuals over 18 years of age and is also administered in two doses, with a recommended internal of 4–12 weeks after the first dose [5,17]. The Ad26.COV2.S vaccine is a recombinant, replication-incompetent adenovirus type 26 vector vaccine, constructed to encode the S protein. It is administered as a single intramuscular injection to individuals over 18 years of age [5,18].

Previous studies have reported the efficacy of the aforementioned vaccines in the prevention of COVID-19 morbidity and mortality [5,19,20]; however, comparisons in terms of their efficacy and tolerance in the “real world” are rather limited. Therefore, the aim of our study was to record side-effects and compare the efficacy of the vaccines in age- and sex-matched individuals that were randomly selected from the community. Vaccines efficacy was assessed against circulated variants during sample collection period. In this context, we also explored if the intensity of antibody responses was important for the development of COVID-19 following vaccination. We consider that our results may have significant implications on public health vaccination strategies.

## 2. Materials and Methods

### 2.1. Subjects

A total of 270 individuals were enrolled in the study from December 2020 to November 2021. Among these individuals, 135 were vaccinated with adenovirus vector-based vaccines (men/women: 81/54, median age: 49.0 years, range: 20.0–84.0); 67 individuals were vaccinated with two doses of ChAdOx1 nCoV-19 vaccine (AZD1222 of Oxford/AstraZeneca, University of Oxford, UK) (men/women; 39/28, median age: 64.0 years, range: 22.0–84.0) with an interval of 55–96 days, and 68 individuals were vaccinated with a single dose of Ad.COV2.S COVID-19 vaccine (Janssen Biotech, Inc., Janssen Pharmaceutical company, Johnson and Johnson, New Jersey, USA) (men/women: 42/26, median age: 46.5 years, range: 20.0–74.0). The humoral immune responses after adenovirus vector-based vaccines were compared with humoral responses in 135 age- and sex-matched individuals (men/women: 81/54, median age: 49.0 years, range: 27.0–84.0) receiving the BNT162b2 vaccine (Comirnaty^®^; BioNTech/Pfizer) in two doses with an interval of 21 days. Comparisons of antibody responses were performed between individuals of the entire group of viral vector vaccines and the entire group of mRNA BNT162b2 vaccinated participants, as well as between each group of viral vector vaccines and groups of BNT162b2 vaccinated individuals, who were age- and sex-matched.

A total of 17 individuals from the group of mRNA BNT2b2 vaccines and 10 from the Ad26.COV2.S COVID-19 vaccine exhibited a positive history of COVID-19 prior to vaccination. In addition, anti-N IgG antibodies were assessed as an indicator of recent SARS-CoV-2 infection, and positive titers were detected at enrollment in eight additional participants without a known history of COVID-19 infection.

Serum sampling was performed in all vaccinated individuals at (a) day 21, (b) day 42, (c) day 90, and (d) day 180 following the first dose of vaccination. During the study, participants were asked to complete a questionnaire regarding their medical history, adverse reactions after each dose of vaccination, and possible post-vaccination infection with SARS-CoV-2. Table 1 presents a detailed overview of participants’ medical history, including a history of COVID-19 before vaccination.

Each participant or their relatives, in the case of insomnia or mental disorders, provided signed informed consent. The study was conducted based on the principles of the Helsinki Declaration and was approved by the Ethical Committee of the Faculty of Medicine, University of Thessaly, Greece (No 2116).

### 2.2. Laboratory Tests

Anti-S and anti-N anti-SARS-CoV-2 IgG and IgA antibodies were quantified by chemiluminescent microparticle immunoassay (CMIA) and enzyme linked immunosorbent assay (ELISA), as described [21,22].

### 2.3. Statistical Analysis

Categorical variables are described using frequencies and relative frequencies, while continuous variables are described with medians and interquartile ranges (IQRs). The analysis of continuous variables was conducted using the Mann–Whitney U test and Spearman’s correlation coefficient, since the assumption of normal distribution was violated. Data were checked for deviation from normal distribution using the Shapiro–Wilk normality test. Multivariate analysis was performed in the form of multiple regression and binary logistic regression. Multiple regression was used to determine independent predictors of antibodies’ quantity/levels, and binary logistic regression was used to determine independent predictors of infection. For all analyses, a 5% significance level was set. Analysis was carried out with IBM Corp. Released 2019 - IBM SPSS Statistics for Windows, Version 26.0 (IBM Corp., Armonk, NY: IBM Corp, USA) and GraphPad Prism for Windows, GraphPad Software, San Diego, California USA (version 9.2.0).

## 3. Results

### 3.1. Adverse Reactions after Vaccination

A total of 268 out of 270 participants (99.3%) were fully vaccinated according to the initial vaccination schedule. Two individuals did not receive the second dose; this included a 58 year old female from the BNT162b2 group due to severe facial flushing and electrocardiogram (ECG) changes and a 63 year old female from the ChAdOx1 nCoV-19 group due to tachycardia, facial flushing, and reactivation of herpes zoster. Complete records of all side-effects reported after each dose of vaccine were available for 239 individuals (89.2%), while, for the 31 remaining participants, the only information available was related to the presence of fever.

An overview and a comparison of the most common side-effects after vaccination are presented in Table 2 and Table 3, respectively. The presence of local pain—either with or without redness and swelling—at the injection site was the most common side-effect after the first dose of BNT162b2, compared to the Ad26.COV2.S vaccine (Table 2). Fever occurred more frequently in adenovirus vector-based vaccines (Table 2 and Table 3). Moreover, myalgias were reported more frequently after the second dose of BNT162b2 vaccine when compared to the Ad26.COV2.S group, as well as following the first dose of ChAdOx1 nCoV-19 vaccine in comparison to the BNT162b2 group. Reported side-effects were referred to as mild and limited in all study participants, except for two cases that did not receive the second dose as mentioned above (Table 2 and Table 3).

### 3.2. Intensity and Dynamics of IgG and IgA Responses after Vaccination

**Figure 1:**
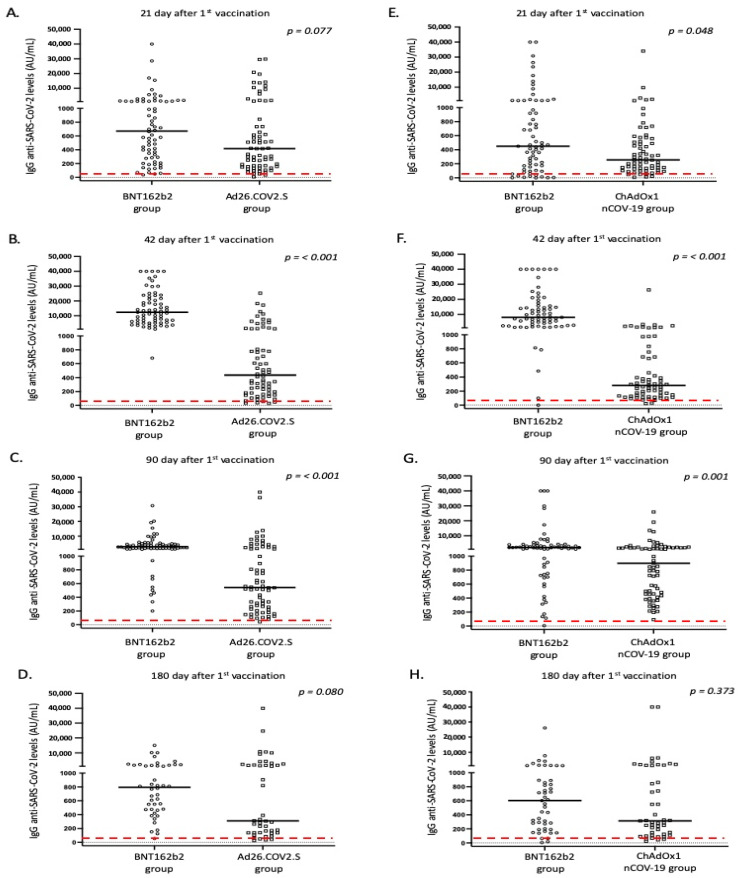
IgG anti SARS-CoV-2 responses in the study participants according to vaccine platform: (**A**,**E**) day 21, (**B**,**F**) day 42, (**C**,**G**) day 90, and (**D**,**H**) day 180. Black lines indicate median values and red dotted lines represent the cutoff of positive anti-SARS-CoV-2 IgG (50 AU/mL) antibodies.

**Figure 2:**
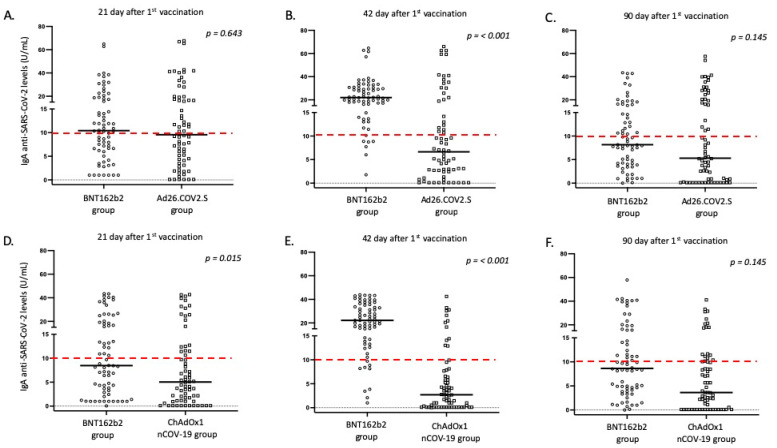
IgA anti SARS-CoV-2 responses in the study participants according to vaccine platform: (**A**,**D**) day 21, (**B**,**E**) day 42, and (**C**,**F**) day 90. Black lines indicate median values, and red dotted lines represent the cutoff of positive anti-SARS-CoV-2 IgA (10 U/mL) antibodies.

All platforms achieved positive IgG antibody responses for the great majority of vaccinated individuals 21 days after vaccination; however, the intensity of responses differed (Table 4, Figure 1 and Figure 2). Specifically, the BNT162b2 vaccine resulted in significantly higher antibody titers when compared to the total number of adenovirus vector-based vaccines (*p* < 0.001) and to ChAdOx1nCoV-19 (*p* = 0.048). While differences were also present when BNT162b2 was compared with the Ad26.COV2.S vaccine, this difference was not considered significant (*p* = 0.077). In terms of IgA responses, most enrolled individuals failed to respond adequately (with antibody titers below the threshold of positivity); nevertheless, the intensity of IgA titers was higher after BNT162b2 vaccination (Table 4 and Figure 2).

Furthermore, on the 42nd day after the first dose, individuals who received the second dose of the mRNA vaccine displayed a significantly higher proportion of positive anti-S IgG and IgA antibodies, when compared to adenovirus vector-based vaccines (*p* < 0.001). These individuals also exhibited significantly higher levels of IgG and IgA anti-SARS-CoV-2 antibodies in their serum (Table 4 and Figure 1 and Figure 2). This pattern of expression was maintained for IgG responses at 90 days following the first vaccination dose, but was missed for IgA responses, where the majority of immunized individuals displayed IgA levels below the positivity cutoff (Table 4 and Figure 1 and Figure 2).

Unfortunately, not all individuals participated in blood sampling on day 180 following the first dose. Those who remained in the study (182 out of 270, 67.4%) continued to be age- and sex-matched. Interestingly, although anti-S IgG levels were higher in the BNT162b2 group, the statistical significance was marginally lost (especially comparing IgG levels between BNT162b2 and Ad26.COV2.S vaccines; Table 4, Figure 1). As previously mentioned, considering that most enrolled individuals displayed very low IgA levels on day 90, we did not measure IgA responses on day 180.

### 3.3. Correlation of IgG Responses with Demographic and Clinical Parameters of Vaccinated Individuals

According to multivariate analysis (Table 5) which included data from individuals vaccinated with all three vaccines, the main factors affecting IgG levels following vaccination were a history of COVID-19 infection (prior to or after vaccination) and the vaccine platform. Specifically, BNT162b2 vaccination was associated with higher IgG titers on sampling days 42 and 90 after vaccination, compared to adenovirus vector-based vaccines (*p* < 0.001 and *p* = 0.001, respectively). Moreover, SARS-CoV-2 infection confirmed by RT-PCR or detection of positive anti-N anti-SARS-CoV-2 antibodies was the constant factor with a positive effect on IgG antibody levels; no other factors from participants’ medical histories affected the intensity of antibody responses. When participants with a history of COVID-19 infection were excluded from analysis, the vaccine platform was the predominant factor associated with IgG antibody responses on sampling day 21 (*p* = 0.002), day 42 (*p* < 0.001), and day 90 (*p* = 0.002) following the first dose, with the BNT162b2 vaccine appearing superior compared to adenovirus vector-based vaccines. Age significantly affected the antibody titer only on sampling day 42 and was associated with lower IgG levels among older individuals. Considering the effect of adverse reactions on intensity of IgG responses, multivariate analysis revealed that the presence of fever after both doses, and fatigue after the second dose was associated with higher IgG antibody titers (Supplementary Table S1). When participants with a known history of COVID-19 were excluded from analysis, no side-effects appeared to have an effect on antibody responses.

By studying vaccine platforms in pairs, similar data were provided through multivariate analysis of IgG responses. In particular, previous COVID-19 infection always had a positive effect on the intensity of IgG responses; however, when participants with a known infection history were excluded from analysis, the vaccine platform played a significant role, which was more profound when compared to individuals receiving BNT162b2 and Ad26.COV2.S vaccines on sampling days 21, 42, and 90 (Supplementary Table S2). Further analysis revealed that participants receiving antihypertensive treatment and Ad26.COV2.S vaccine, displayed lower anti-SARS-CoV-2 levels on sampling day 42 (*p* = 0.019) (Supplementary Table S3). Lastly, individuals displaying headaches exhibited higher IgG levels on day 42, when compared to the BNT162b2 and ChAdOx1 nCoV-19 groups (*p* = 0.019).

### 3.4. Correlation of IgA Responses with Demographic and Clinical Parameters of Vaccinated Individuals

Similar to anti-S IgG responses, IgA levels were significantly affected by COVID-19 history before or after vaccination (*p* < 0.001). Multivariate analysis revealed that the type of vaccination significantly affected IgA levels on sampling days 42 and 90 following the first dose, regardless of COVID-19 history prior to or after vaccination (Table 6). Lastly, adverse side-effects did not affect the intensity of IgA responses, except for the presence of fever after the second immunization with BNT162b2 vaccine, especially when compared to BNT162b2 and ChAdOx1 nCoV-19 groups (*p* = 0.011).

### 3.5. Correlation of Antibody Titers and COVID-19 after Vaccination

A total of 18 participants (including two with prior SARS-CoV-2 infection, one from the BNT162b2 group, three from the ChAdOx1 nCoV-19 group, and 14 from the Ad26.COV2.S group) were infected by SARS-CoV-2 after vaccination, as confirmed by detection of the virus via RT-PCR on nasopharyngeal swabs or, in one case, by the detection of anti-N anti-SARS-CoV-2 antibodies in serum. The confirmed COVID-19 cases were distributed at different sampling times. Specifically, two participants were infected by SARS-CoV-2 between the 21st and 42nd day after the first dose, five between the 42nd and 90th day, and 11 between the 90th and 180th day. All COVID-19 patients displayed mild disease, and none required hospitalization.

**Figure 3:**
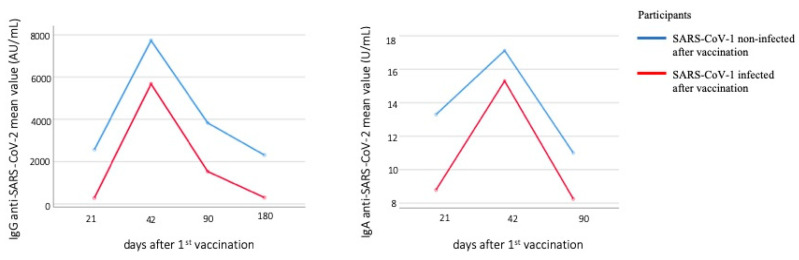
Line chart of anti-S IgG and IgA antibodies levels between infected and noninfected with SARS-CoV-2 after vaccination individuals.

A line chart was used to represent levels of anti-S IgG and IgA antibodies over time between infected and noninfected groups. It appears that those who were infected with SARS-Cov-2 had lower antibody titers throughout the study [23] (Figure 3).

## 4. Discussion

Our study provides clear evidence that both mRNA and adenovirus vector-based vaccines are effective for inducing adequate antibody responses against SARS-CoV-2, while also demonstrating that the BNT162b2 mRNA vaccine is superior to adenovirus vector-based vaccines concerning the strength of antibody responses and protection against severe COVID-19.

As previously mentioned, all vaccine platforms were well tolerated, and side-effects were limited for most enrolled participants except two females, reported above, which did not receive a second dose due to severe adverse reactions. Similar to previous studies [24,25,26,27,28], local side-effects were more common after vaccination, while systemic adverse reactions (mild as a rule) were more common following adenovirus vector-based vaccines. Despite the presence of mild side-effects in the majority of individuals of our study, the number remains small to draw conclusions about the safety of vaccine platforms.

In our study, we compared the intensity of antibody responses between individuals receiving mRNA and adenovirus vector-based vaccines. Although similar studies have been published in the literature [29,30], our study has two significant differences which may represent important advantages. The first is that we compared antibody responses of age- and sex-matched participants from the community, while most previous studies compared only healthcare workers. The second was the inclusion of IgA response intensity as a significant parameter of immunization.

Both we and others have already reported that anti-S IgA responses after mRNA vaccination display a rapid decline compared to IgG responses, particularly 3 months following vaccination [25,31,32,33]. Although Zurac et al. reported a higher rate of positive IgA responses after vaccination, similar to our results, IgA levels were positively correlated only with a previous history of COVID-19 [32]. It is worth noting that IgA responses were evaluated in previous studies especially after mRNA vaccination, including a very small number of participants. To the best of our knowledge, our study is the largest in the literature analyzing IgA responses after vaccination with different vaccine platforms, and we identified that BNT162b2 vaccination resulted in the strongest positive effect on IgA antibody titers.

As with previous studies, we demonstrated that the BNT62b2 mRNA vaccine resulted in higher titers of anti-IgG levels compared to adenovirus vector-based vaccines [29,30,33,34]. In addition, serological data after the second dose of BNT162b2 vaccine resulted in boosting of anti-S IgG antibody titers, with a further waning over time [29]. Similar to previous studies, we also observed that age and a previous history of COVID-19 significantly affected intensity of IgG responses [22,29,35,36].

Interestingly, we identified that individuals immunized with BNT62b2 vaccine who displayed higher anti-S IgA and IgG antibody levels exhibited a lower incidence of COVID-19 after vaccination, compared to those immunized with adenovirus vector-based vaccines. In particular, 14 individuals (out of a total of 18 infected by SARS-CoV-2 after vaccination) were immunized with the Ad26.COV2.S vaccine, compared to three with the ChAdOx1 nCoV-19 vaccine and only one with the BNT162b2 vaccine. As mentioned above, the effectiveness of COVID-19 vaccination slowly decreases over time, which is more profound at 6 months following vaccination [29,37]. This observation is in line with Cohn et al., where protection against severe SARS-CoV-2 for individuals who received the BNT162b2 vaccine decreased from 87% to 45% 6 months after vaccination, while protection against severe SARS-CoV-2 for individuals who received the Ad26.COV2.S vaccine dropped from 86% to 13% [38]. Therefore, we could speculate that lower antibody levels, faster waning of antibody titers over time, and a higher rate of SARS-CoV-2 infection after Ad26.COV2.S immunization lead to the conclusion that a single-dose vaccine is less effective than others.

A limitation of our study is the relatively small number of enrolled participants. However, we conducted a real-world study in the community; a significant challenge was enrolling age- and sex-matched participants, considering the different politics concerning vaccination strategies among various age populations. For example, according to initial data of thrombotic events after adenovirus vector-based vaccines, this type of vaccination platform was contraindicated for women below 60 years of age and young individuals for a long period of time [39,40]. Moreover, it is always challenging to convince individuals who are not health workers to provide a blood donation. Thus, selecting individuals of different age groups who were age- and sex- matched was rather difficult, but we consider the number of enrolled individuals as adequate to provide conclusive remarks.

## 5. Conclusions

Our study provides supportive evidence about the effectiveness of COVID-19 vaccination. We identified that anti-S IgG and IgA responses depend primarily on both the presence of a previous COVID-19 infection history and the vaccination platform used, with the mRNA vaccine appearing superior to adenovirus vector-based vaccines. Furthermore, individuals who became infected following vaccination had lower antibody titers and were mainly from the Ad26.COV2.S group, suggesting that a single-dose vaccine is less effective than the other vaccines. We consider that our results will be useful for the implementation of further vaccination strategies.

## Figures and Tables

**Table 1:** Overview of demographic and clinical data of SARS-CoV-2 vaccinated participants.

	BNT162b2	ChAdOx1 nCoV-19	Ad26.COV2.S
	135 (*n*, %)	67 (*n*, %)	68 (*n*, %)
Gender (male/female)	81/54	39/28	42/26
Age (years; median, range)	49.0 (27–84)	64.0 (33–84)	46.5 (20–74)
Hypertension	29, 21.5	30, 44.8	6, 8.8
Diabetes mellitus	11, 8.2	11, 16.4	1, 1.5
Dyslipidemia	23, 17.0	20, 29.9	2, 2.9
Chronic heart disease ^	9, 6.7	6, 9.0	3, 4.4
Chronic respiratory disease *	5, 3.7	1, 1.5	1, 1.5
Stroke and transient ischemic attacks	7, 5.2	1, 1.5	0, 0.0
Thrombotic attacks	0, 0.0	0, 0.0	2, 2.9
Chronic liver disease #	2, 1.5	1, 1.5	0, 0.0
Chronic kidney disease ^^	1, 0.7	0, 0.0	0, 0.0
Thyroid disease **	10, 7.4	6, 9.0	5, 7.4
Autoimmune/autoinflammatory disease §	7, 5.2	2, 3.0	0, 0.0
Cancer ##	2, 1.5	1, 1.5	1, 1.5
Insomnia or psychiatric diseases ***	25, 18.5	1, 1.5	2, 2.9
Others ^^^	20, 14.8	10, 14.9	3, 4.4
Previous COVID-19 disease	20, 14.8	2, 3.0	13, 19.1

^ Chronic heart disease includes atrial fibrillation, arrhythmias, myocardial infraction, myocarditis, and coronary heart disease; * chronic respiratory disease includes chronic obstructive pulmonary disease, and asthma; # chronic liver disease includes primary biliary cirrhosis and alcohol-related liver disease; ^^ chronic kidney disease includes chronic renal insufficiency; ** thyroid disease includes hypothyroidism (non-autoimmune) and Hashimoto disease; § autoimmune/autoinflammatory disease includes scleroderma, sarcoidosis, psoriasis and psoriatic arthritis, lupus, Henoch–Schoenlein purpura, polymyalgia rheumatica, and myasthenia gravis; ## cancer includes patients with history of thyroid and testicular cancer and hematologic malignancies (leukemia); *** insomnia or psychiatric diseases includes patients with insomnia, schizophrenia, depression, bipolar disorders, and psychosis; ^^^ other diseases include prostate hyperplasia, vision and hearing disorders, chronic alcohol use, Down syndrome, osteoporosis, spastic quadriplegia, history of epilepsy, anemia, pyramidal tract disorders, hyperuricemia, Parkinson’s disease, pituitary microadenoma, and stomach ulcer.

**Table 2:** Comparison of adverse reactions after vaccination with BNT162b2 and Ad26.COV2.S vaccines.

	BNT162b2 (No. 68)		Ad26.COV2.S (No. 68)	*p*1 *^*	*p*2 *^*
	1st Dosage (*n*, %)	2nd Dosage (*n*, %)	1st Dosage (*n*, %)		
Sex (M/F)	42/26		42/26		
Age (years; median, range)	45.0, 27–74		46.5, 20–74		
Local pain	22, 34.9	15, 23.8	12, 17.6	0.029	0.397
Fever	1, 1.5	9, 13.2	20, 29.4	<0.001	0.035
Myalgias	3, 4.8	13, 20.6	4, 5.9	1.000	0.018
Fatigue	4, 6.4	15, 23.8	14, 20.6	0.022	0.679
Headache	6, 9.5	2, 3.2	7, 10.3	1.000	0.167
Flu-like symptoms	0, 0.0	1, 1.6	0, 0.0	1.000	0.481
Others *	4, 6.4	7, 11.1	8, 11.8	0.369	1.000

* Other symptoms include chills (four participants), nausea (four), vertigo (three), numbness (two), drowsiness (two), cough (one), lymphadenitis (one), thorax pain and backache (one), malaise (one), taste disorders (one), sore throat and swelling (one), and urticaria (one). ^ *p*1 refers to the comparison of two vaccines after the first dose, and *p*2 refers to the comparison of second dose of BNT162b2 with Ad26.COV2.S vaccines.

**Table 3:** Comparison of adverse reactions after vaccination with BNT162b2 and ChAdOx1 nCoV-19 vaccines.

	**BNT162b2** **(No. 67)**		**ChAdOx1 nCoV-19** **(No. 67)**		***p*1 *^***	***p*2 *^***
	**1st Dosage (*n*, %)**	**2nd Dosage** **(*n*, %)**	**1st Dosage** **(*n*, %)**	**2nd Dosage** **(*n*, %)**		
Sex (M/F)	39/28	39/27	39/28	39/27		
Age (years; median, range)	64.0 (33–84)		64.0 (33–84)			
Local pain	10, 24.4	8, 20.0	21, 31.3	11, 16.7	0.514	0.795
Fever	1, 1.5	3, 4.6	18, 26.9	3, 4.6	<0.001	1.000
Myalgias	0, 0.0	2, 5.0	8, 11.9	2, 3.0	0.023	0.632
Fatigue	4, 9.8	7, 17.5	15, 22.4	6, 9.1	0.121	0.231
Headache	3, 7.3	3, 7.5	7, 10.5	1, 1.5	0.739	0.149
Flu-like symptoms	0, 0.0	0, 0.0	1, 1.5	0, 0.0	1.000	1.000
Others *	1, 2.4	0, 0.0	8, 11.9	7, 10.6	0.149	0.043

* Other symptoms include chills (six participants), vertigo (four), flushing (two), tachycardia (two), ECG changes (one), reactivation of Herpes Zoster (one), and drowsiness (one). ^ *p*1 refers to the comparison of adverse reactions of the first dose, and *p*2 refers to the comparison of adverse reactions of the second dose of both vaccines.

**Table 4:** IgG and IgA immune responses 21, 42, 90, and 180 days after vaccination.

	BNT162b2 (No. 67)	ChAdOx1 nCoV-19H (No. 67)	*p*1 *	BNT162b2 (No. 68)	Ad26.COV2.S (No. 68)	*p*2 *	BNT162b2 Total	Adenovirus Vector-Based Total	*p*3 *
Sex (male/female)	39/28	39/28		42/26	42/26		81/54	81/54	
Age (median, range)	64.0, 33.0–84.0	64.0, 33.0–84.0		45.0, 27.0–74.0	46.5, 20.0–74.0		49.0, 27.0–84.0	49.0, 20.0–84.0	
Day 21									
IgG (median, range) (AU/mL)	450.5, 0.0–40,000.0	255.4, 7.8–33,994.22	0.048	670.8, 35.9–40,000.0	416.2, 7.1–29,806.1	0.077	541.8, 0.0–40,000.0	308.2, 7.1–33,994.2	<0.001
IgG positivity (*n*, %)	58, 86.6	63, 94.0		67, 98.5	64, 94.1		125, 92.6	128, 94.8	
IgA median, range (U/mL)	8.5, 0.0–43.5	5.0, 0.1–42.7	0.015	10.4, 1.0–64.9	9.6, 0.1–67.6	0.643	10.3, 14.2, 0.0–64.9	7.2, 0.1–67.6	0.023
IgA positivity (*n*, %)	31, 46.3	19, 28.4		37, 54.4	33, 48.5		68, 50.4	52, 38.5	
Day 42									
IgG (median, range) (AU/mL)	8015.3, 0.2–40,000.0	281.0, 22.4–26,246.5	<0.001	12,369.4, 680.8–40,000.0	436.5, 29.6–25,236.4	<0.001	10,576.2, 0.2–40,000.0	326.9, 22.4–26,246.5	<0.001
IgG positivity (*n*, %)	66, 98.5	65, 97.0		68, 100.0	66, 97.1		134, 99.3	131, 97.0	
IgA median, range (U/mL)	22.2, 1.0–43.9	2.7, 0.0–42.5	<0.001	22.0, 1.8–64.6	6.7, 0.1–66.0	<0.001	22.2, 1.0–64.6	4.2, 0.0–66.0	<0.001
IgA positivity (*n*, %)	59, 88.10	11, 16.4		62, 91.2	24, 35.3		121, 89.6	35, 25.9	
Day 90									
IgG (median, range) (AU/mL)	2181.9, 5.4–40,000.0	898.1, 91.8–25,934.9	0.001	2822.2, 200.5–30,759.0	543.6, 43.2–40,000.0	<0.001	2345.4, 5.4–40,000.0	745.3, 1 43.2–40,000.0	<0.001
IgG positivity (*n*, %)	66, 98.5	67, 100.0		68, 100.0	67, 98.5		134. 99.3	134, 99.3	
IgA median, range (U/mL)	8.8, 0.0–57.9	3.6, 0.1–41.1	0.145	8.2, 0.0–43.4	5.3, 0.0–57.5	0.145	8.6, 1 0.0–57.9	4.7, 0.1–57.5	<0.001
IgA positivity (*n*, %)	29, 43.3	17, 25.4		29, 42.6	26, 38.2		58, 43.0	43, 31.9	
Day 180	(n: 47)			(n: 44)			(n: 91)		
IgG (median, range) (AU/mL)	602.5, 5.0–26,031.9	314.2, 23.3–40,000.0	0.373	795.1, 55.2–14,969.0	309.8, 25.2–40,000.0	0.080	686.7, 5.0–26,031.9	312.4, 23.3–40,000.0	0.057
IgG positivity (*n*, %)	45, 95.7	45, 95.7		44, 100.0	40, 90.9		89, 97.8	85, 93.0	

* *p*1 refers to the comparison of BNT162b2 and ChAdOx1 nCoV-19, *p*2 refers to the comparison of BNT162b2 and Ad26.COV2.S, and *p*3 refers to the comparison of BNT162b2 and adenovirus vector-based vaccines.

**Table 5:** Multivariate analyses of anti-SARS-CoV-2 IgG responses after vaccination with adenovirus vector-based (Ad26.COV2.S, ChAdOx1 nCoV-19) and mRNA (BNT162b2) vaccines.

Dependent Variable	Parameter	*p*1	Coefficient	*p*2 *	Coefficient
Anti-S IgG levels on day 21	Age	0.611	12.2 (−34.9, 59.3)	0.079	−9.4 (−20.0, 1.1)
	Vaccine type (adenovirus vector-based vs. mRNA)	0.484	−381.5 (−1452.6, 689.7)	0.002	−381.3 (−618.9, −143.8)
	Comorbidity (no vs. ≥1)	0.472	495.9 (−859.9, 1851.7)	0.098	−248.1 (−542.6, 46.4)
	COVID-19 history before vaccination	<0.001	15,364.8 (13,768.0, 16,961.6)	Excluded	
Anti-S IgG levels on day 42	Age	0.441	−31.65 (−112.38, 49.10)	0.009	−97.89 (−171.36, −24.41)
	Vaccine type (adenovirus vector-based vs. mRNA)	<0.001	−12,041 (−13,875.5, −10,206.4)	<0.001	−10,707.98 (−12,356.9, 9059.0)
	Comorbidity (no vs. ≥1)	0.358	−1082.2 (−3396.3, 1232.0)	0.077	−1835.1 (−3872.8, 202.6)
	COVID-19 history before vaccination	<0.001	12,559.8 (9832.2, 15,287.5)	Excluded	
	COVID-19 history after vaccination	0.002	17,143.2 (−6431.5, 27,855.0)		
Anti-S IgG levels on day 90	Age	0.103	48.12 (−9.78, 106.02)	0.268	−18.6 (−51.49, 14.38)
	Vaccine type (adenovirus vector-based vs. mRNA)	0.001	−2320.6 (−3635.8, −1005.3)	0.002	−1149.5 (−1888.9, −410.1)
	Comorbidity (no vs. ≥1)	0.590	453.6 (−1202.7, 2110.0)	0.665	200.2 (−709.8, 1110.3)
	COVID-19 history before vaccination	<0.001	8871.6 (6923.1, 10,820.1)	Excluded	
	COVID-19 history after vaccination	<0.001	16,889.1 (12,695.6, 21,082.6)		
Anti-S IgG levels on day 180	Age	0.522	20.54 (−42.71, 83.79)	0.077	−14.18 (−29.92, 1.55)
	Vaccine type (adenovirus vector-based vs. mRNA)	0.542	−435.8 (−1844.6, 973.0)	0.204	−222.8 (−567.8, 122.1)
	Comorbidity (no vs. ≥1)	0.564	504.7 (−1219.2, 2228.6)	0.840	−43.4 (−466.9, 380.2)
	COVID-19 history before vaccination	0.003	3419.2 (1162.7, 5675.7)	Excluded	
	COVID-19 history after vaccination	<0.001	14,638.1 (11,790.0, 17,486.3)		

* *p*2 refers to multivariate analysis excluding the parameter of the history of COVID-19 prior and after vaccination.

**Table 6:** Multivariate analyses of anti-SARS-CoV-2 IgA responses after vaccination with adenovirus vector-based (Ad26.COV2.S, ChAdOx1 nCoV-19) and mRNA (BNT162b2) vaccines.

Dependent Variable	Parameter	*p*1	Coefficient	*p*2 *	Coefficient
Anti-S IgA levels on day 21	Age	0.146	−0.08 (−0.19, 0.03)	0.301	−0.05 (−0.16, 0.05)
	Vaccine type (adenovirus vector-based vs. mRNA)	0.551	−0.7 (−3.2, 1.7)	0.093	−2.0 (−4.3, 0.3)
	Comorbidity (no vs. ≥1)	0.560	−0.9 (−4.0, 2.2)	0.245	−1.7 (−4.6, 1.2)
	COVID-19 history before vaccination	<0.001	27.8 (24.1, 31.3)	Excluded	
Anti-S IgA levels on day 42	Age	0.186	−0.08 (−0.19, 0.04)	0.651	−0.03 (−0.14, 0.09)
	Vaccine type (adenovirus vector-based vs. mRNA)	<0.001	−14.2 (−16.8, −11.6)	<0.001	−16.1 (−18.6, −13.6)
	Comorbidity (no vs. ≥1)	0.520	−1.1 (−4.4, 2.2)	0.301	−1.6 (−4.7, 1.5)
	COVID-19 history before vaccination	<0.001	22.4 (18.5, 26.2)	Excluded	
	COVID-19 history after vaccination	0.001	26.3 (10.9, 41.6)		
Anti-S IgA levels on day 90	Age	0.661	0.02 (−0.08, 0.12)	0.765	0.01 (−0.08, 0.11)
	Vaccine type (adenovirus vector-based vs. mRNA)	0.009	−2.9 (−5.2, −0.8)	0.002	−3.2 (−5.2, −1.2)
	Comorbidity (no vs. ≥1)	0.680	−0.6 (−3.3, 2.2)	0.280	−1.4 (−3.9, 1.1)
	COVID-19 history before vaccination	<0.001	22.5 (19.2, 25.7)	Excluded	
	COVID-19 history after vaccination	<0.001	21.4 (13.8, 28.9)		

* *p*2 refers to multivariate analysis excluding the parameter of the history of COVID-19 prior and after vaccination.

## Data Availability

The data that support the findings of this study are available from the corresponding author upon request.

## Supplementary Materials

The following supporting information can be downloaded at https://www.mdpi.com/article/10.3390/vaccines10081268/s1: Table S1. Multivariate analyses of adverse side-effects and intensity of anti-SARS-CoV-2 IgG responses after vaccination with adenovirus vector-based (Ad26.COV2.S, ChAdOx1 nCoV-19) and mRNA (BNT162b2) vaccines on day 42 after vaccination; Table S2. Multivariate analyses of anti-SARS-CoV-2 IgG responses after vaccination with Ad26.COV.2.S and BNT162b2 vaccines; Table S3. Multivariate analysis of intensity of anti-SARS-CoV-2 IgG responses on sampling day 42 after the first dose of BNT162b2 and Ad26.COV2.S vaccination.
