# Supplementary material for: Intensity of Humoral Immune Responses, Adverse Reactions, and Post-Vaccination Morbidity after Adenovirus Vector-Based and mRNA Anti-COVID-19 Vaccines
Source: Vaccines (Basel). 2022 Aug 6;10(8):1268. doi: 10.3390/vaccines10081268 (PMC9416671; doi:10.3390/vaccines10081268)
Supplement: Supplementary file 1 [file vaccines-10-01268-s001.zip › Supplementary Table S1.pdf]

Supplementary table S1. Multivariate analyses of adverse side effects and intensity of anti-SARS-CoV-2 IgG responses after vaccination with adenovirus vector-based (Ad26.COV2.S, ChAdOx1 nCoV-19) and mRNA (BNT162b2) vaccines on day 42 after vaccination

Parameter	N, median IgG levels (mg/dL) (IQR)	*p*1	Coefficient, 95% CI	*p*2*	Coefficient, 95% CI
Age (years)	rho=-0.187	0.577	-32.1 (-145.3 , 81.2)	0.095	-89.8 (-195.5 , 16.0)
Vaccination	BNT162b2:135, 10576.2 (15420.4) Ad26.COV2.S: 68, 436.5 (1095.7) ChAdOx1 nCoV-19: 67, 281.0 (552.3)	<0.001	(Ad26.COV2.S, ChAdOx1 nCoV-19 / BNT162b2) -10203.6 (-12729.4 , -7687.8)	<0.001	(Ad26.COV2.S, ChAdOx1 nCoV-19 / BNT162b2) -9328.1 (-11636.9 , -7019.2)
Comorbidity (no vs ≥ 1)	≥ 1: 134, 1682.6 (8096.1) No:136, 3632.8 (13075.0)	0.555	-895.9 (-3886.7 , 2095.0)	0.265	-1584.3 (-4383.0 , 1214.3)
Localpain_2	Yes: 34, 9192.6 (14174.9) No: 135, 3893.2 (12751.8)				
Fever_2	Yes: 15, 20378.3 (34830.1) No:185, 4523.6 (12837.2)	0.005	6297.6 (1981.2 , 10614.1)	0.070	4075.6 (-330.6 , 8481.7)
Myalgias_2	Yes:17, 17718.2 (14188.6) No: 152, 3545.3 (11935.7)	0.482	1705.1 (-3069.3 , 6479.6)	0.187	3171.8 (-1560.5 , 7904.1)
Fatigue_2	Yes:28, 14674.9 (20022.0) No:141, 3267.5 (10941.2)	0.049	3780.4 (17.2, 7543.6)	0.141	2630.0 (-868.1 , 6146.1)
Headache_2	Yes: 6, 10344.8 (13898.1) No: 163, 4957.9 (13155.9)				
Flulike_2	Yes: 1 No: 168, 4980.1 (13385.7)				
Others_2	Yes: 14, 1865.7 (16647.8) No: 155, 5169.9 (13438.9)				
COVID-19 history (before vaccination)	Yes:35, 15665.4 (34529.1) No:235, 1316.1 (9022.3)	<0.001	16368.7 (12373.4 , 20363.9)	Excluded	
COVID 19 history (after vaccination)	Yes:2, 18226.0 (-) No:268, 2155.7 (11214.2)	-	-		

* *p*2 refers to multivariate analysis excluding the parameter of the history of COVID-19 prior and after vaccination
