# Supplementary material for: Intensity of Humoral Immune Responses, Adverse Reactions, and Post-Vaccination Morbidity after Adenovirus Vector-Based and mRNA Anti-COVID-19 Vaccines
Source: Vaccines (Basel). 2022 Aug 6;10(8):1268. doi: 10.3390/vaccines10081268 (PMC9416671; doi:10.3390/vaccines10081268)
Supplement: Supplementary file 1 [file vaccines-10-01268-s001.zip › Supplementary Table S2.pdf]

Supplementary Table S2. Multivariate analyses of anti-SARS-CoV-2 IgG responses after vaccination with Ad26.COV2.S and BNT162b2 vaccines

Dependent Variable	Parameter	*p*1	Coefficient	*p*2*	Coefficient
anti–S IgG levels on day 21	Age	0.387	28.42 (-36.40 , 93.24)	0.153	-15.84 (-37.63 , 5.95)
	Vaccine type (Ad26.COV2.S vs BNT162b2)	0.075	-1212.2 (-2547.5 , 123.1)	0.003	-681.8 (-1122.3 , -241.3)
	Comorbidity (no vs ≥ 1)	0.536	491.0 (-1073.4 , 2055.5)	0.180	-351.9 (-868.1 , 164.3
	COVID 19 history before vaccination	<0.001	15937.0 (13937.2 , 17936.8)	Excluded	
anti–S IgG levels on day 42	Age	0.757	-20.60 (-152.20 , 111.0)	0.114	-110.6 (-248.1 , 26.8)
	Vaccine type (Ad26.COV2.S vs BNT162b2)	<0.001	-14830.1 (-17544.0 , -12116.1)	<0.001	-13283.6 (-16032.5 , -10534.7)
	Comorbidity (no vs ≥ 1)	0.388	-1378.8 (-4531.0 , 1773.3)	0.210	-2029.5 (-5220.5 , 1161.6)
	COVID 19 history before vaccination	<0.001	10724.5 (6676.9 , 14772.2)	Excluded	
	COVID 19 history after vaccination	0.002	17966.0 (6825.2 , 29106.7)		
anti–S IgG levels on day 90	Age	0.230	50.86 (-32.60 , 134.31)	0.006	-59.29 (-101.1 , -17.5)
	Vaccine type (Ad26.COV2.S vs BNT162b2)	<0.001	-3116.7 (-4842.7 , -1390.6)	<0.001	-2352.3 (-3198.1 , -1506.5)
	Comorbidity (no vs ≥ 1)	0.673	-429.8 (-2437.2 , 1577.8)	0.336	-475.2 (-1450.4 , 499.9)
	COVID 19 history before vaccination	<0.001	5338.5 (2777.3 , 7899.6)	Excluded	
	COVID 19 history after vaccination	<0.001	24529.8 (10620.8 , 18418.8)		
anti–S IgG levels on day 180	Age	0.294	56.15 (-49.41 , 161.70)	0.659	-16.39 (-90.00 , 57.24)
	Vaccine type (Ad26.COV2.S vs BNT162b2)	0.684	-424.2 (-2488.8 , 1640.4)	0.902	-87.3 (-1315.7 , 1490.4)
	Comorbidity (no vs ≥ 1)	0.489	-789.6 (-3049.2 , 1470.0)	0.261	-868.6 (-2396.9 , 659.7)
	COVID 19 history before vaccination	0.074	2713.7 (-269.6 , 5697.0)	Excluded	
	COVID 19 history after vaccination	<0.001	7857.6 (4381.5 , 11333.7)		

* *p*2 refers to multivariate analysis excluding the parameter of the history of COVID-19 prior and after vaccination
