# Supplementary material for: Intensity of Humoral Immune Responses, Adverse Reactions, and Post-Vaccination Morbidity after Adenovirus Vector-Based and mRNA Anti-COVID-19 Vaccines
Source: Vaccines (Basel). 2022 Aug 6;10(8):1268. doi: 10.3390/vaccines10081268 (PMC9416671; doi:10.3390/vaccines10081268)
Supplement: Supplementary file 1 [file vaccines-10-01268-s001.zip › Supplementary Table S3.pdf]

Supplementary Table S3. Multivariate analysis of intensity of anti-SARS-CoV-2 IgG responses on sampling day 42 after the first dose of BNT162b2 and Ad26.COV2.S vaccination

Parameter	N, median IgG levels (mg/dL) (IQR)	*p*1	Coefficient	*p*2	Coefficient
Sex (male)	Male:84, 4904.5 (12492.3)<br>Female:52, 4164.3, (14772.7)				
Age (years)	rho =-0.184	0.685	27.54 (-106.70 , 161.79)	0.264	-82.4 (-227.8 , 63.0)
Vaccination	Ad26.COV2.S: 68, 436.5 (1095.7)<br>BNT162b2: 68, 12369.4 (17034.2)	<0.001	-14881.7 (-17532.5 , -12230.9)	<0.001	-13363.6 (-16101.1 , -10626.0)
Comorbidity (no vs ≥1)	≥ 1: 37, 3893.2 (11063.0)<br>No:99, 5147.0 (13611.3)				
Comorbidity (0 vs ≥2)	≥ 2:6, 4761.6, (17036.4)<br>No:130, 4865.6 (13394.2)				
Hypertension	Yes:15, 2509.0, (9381.7)<br>No:121, 5080.6, (14324.5)	0.019	-5505.9<br>(-10086.1 , -925.7)	0.095	-3860.8<br>(-8400.0 , 678.4)
Diabetes Mellitus	Yes:5, 7014.2 (18367.4)<br>No:131, 4773.3, (13390.8)				
Dyslipidemia	Yes:7, 7014.2 (10067.3)<br>No:129, 4773.3 (13442.7)				
Chronic heart disease	Yes:7, 7014.2 (9645.6)<br>No:129, 4773.3 (13457.9)				
stroke/ TIA	No:136, 4865.6, (13305.9)				
venous/ arterial thromboses	Yes:2, 2401.5 (-)<br>No:134, 4996.8, (13394.2)				
Chronic respiratory disease	Yes:1<br>No:135, 4957.9, (13364.0)				
Autoimmune/ autoinflammatory disorders	Yes:3, 4957.9 (-)<br>No:133, 4773.3 (13442.7)				
Thyroid disease	Yes:8, 349.8 (24533.8)<br>No: 128, 4996.8 (13251.7)				
Chronic liver disease	No:136, 4865.6, (13305.9)				
Chronic kidney disease	Yes:1, -<br>No:135, 4773.3 (13078.1)				
Cancer	Yes:2, 20074.9<br>No:134, 7424.5 (13136.2)				
Insomnia/ psychiatric diseases	Yes:6, 4865.6 (13394.2)<br>No:130, 3794.8, (9945.8)				
Other	Yes:7, 18802.5 (29765.4)<br>No:129, 4773.3 (12513.6)				
COVID 19 History (before vaccination)	Yes:18, 8767.2 (16179.5)<br>No:118, 3173.6 (12203.3)	<0.001	10439.9 (6465.4 , 14412.7)	Excluded	
COVID 19 History (inf bef42)	Yes:2, 18226 (-)<br>No:134, 4333.3 (13158.7)	0.001	18249.9 (7313.3 , 29186.5)		

* *p*2 refers to multivariate analysis excluding the parameter of the history of COVID-19 prior and after vaccination
